# Supplementary material for: miR 31-3p Has the Highest Expression in Cesarean Scar Endometriosis
Source: Int J Mol Sci. 2022 Apr 22;23(9):4660. doi: 10.3390/ijms23094660 (PMC9105608; doi:10.3390/ijms23094660)
Supplement: Supplementary file 1 [file ijms-23-04660-s001.zip › ijms-1593377-supplementary/Supplementary File S1 - Heat Maps_revised.pdf]

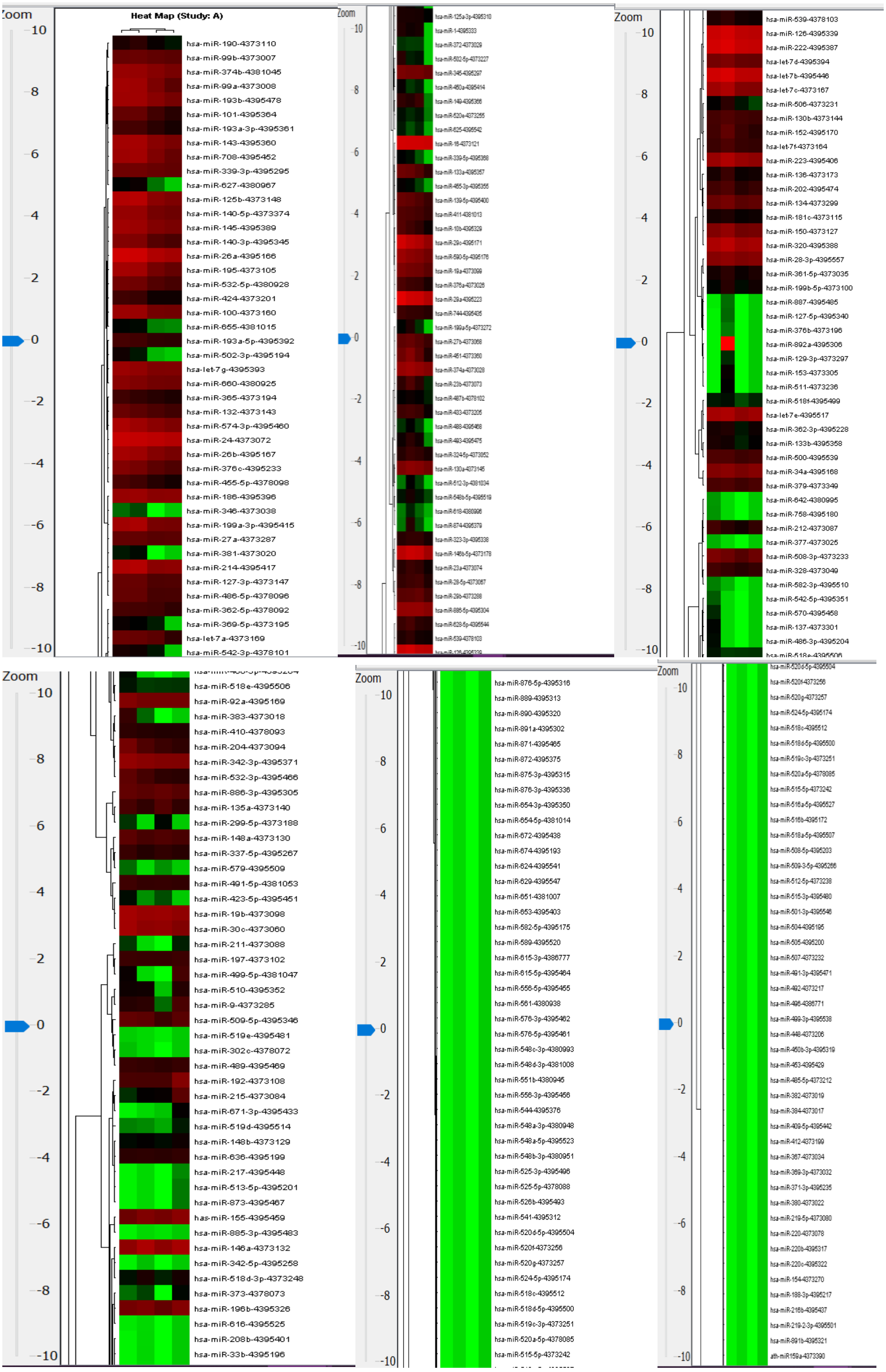
Heat Map (Study: A)
Zoom
hsa-miR-190-4373110
hsa-miR-99b-4373007
hsa-miR-374b-4381045
hsa-miR-99a-4373008
hsa-miR-193b-4395478
hsa-miR-101-4395364
hsa-miR-193a-3p-4395361
hsa-miR-143-4395360
hsa-miR-708-4395452
hsa-miR-339-3p-4395295
hsa-miR-627-4380967
hsa-miR-125b-4373148
hsa-miR-140-5p-4373374
hsa-miR-145-4395389
hsa-miR-140-3p-4395345
hsa-miR-26a-4395166
hsa-miR-195-4373105
hsa-miR-532-5p-4380928
hsa-miR-424-4373201
hsa-miR-100-4373160
hsa-miR-655-4381015
hsa-miR-193a-5p-4395392
hsa-miR-502-3p-4395194
hsa-let-7g-4395393
hsa-miR-660-4380925
hsa-miR-365-4373194
hsa-miR-132-4373143
hsa-miR-574-3p-4395460
hsa-miR-24-4373072
hsa-miR-26b-4395167
hsa-miR-376c-4395233
hsa-miR-455-5p-4378098
hsa-miR-186-4395396
hsa-miR-346-4373038
hsa-miR-199a-3p-4395415
hsa-miR-27a-4373287
hsa-miR-381-4373020
hsa-miR-214-4395417
hsa-miR-127-3p-4373147
hsa-miR-486-5p-4378096
hsa-miR-362-5p-4378092
hsa-miR-369-5p-4373195
hsa-let-7a-4373169
hsa-miR-542-3p-4378101
hsa-miR-539-4378103
hsa-miR-126-4395339
hsa-miR-222-4395387
hsa-let-7d-4395394
hsa-let-7b-4395446
hsa-let-7c-4373167
hsa-miR-506-4373231
hsa-miR-130b-4373144
hsa-miR-152-4395170
hsa-let-7f-4373164
hsa-miR-223-4395406
hsa-miR-136-4373173
hsa-miR-202-4395474
hsa-miR-134-4373299
hsa-miR-181c-4373115
hsa-miR-150-4373127
hsa-miR-320-4395388
hsa-miR-28-3p-4395557
hsa-miR-361-5p-4373035
hsa-miR-199b-5p-4373100
hsa-miR-887-4395485
hsa-miR-127-5p-4395340
hsa-miR-376b-4373196
hsa-miR-892a-4395306
hsa-miR-129-3p-4373297
hsa-miR-153-4373305
hsa-miR-511-4373236
hsa-miR-518f-4395499
hsa-let-7e-4395517
hsa-miR-362-3p-4395228
hsa-miR-133b-4395358
hsa-miR-500-4395539
hsa-miR-34a-4395168
hsa-miR-379-4373349
hsa-miR-642-4380995
hsa-miR-758-4395180
hsa-miR-212-4373087
hsa-miR-377-4373025
hsa-miR-508-3p-4373233
hsa-miR-328-4373049
hsa-miR-582-3p-4395510
hsa-miR-542-5p-4395351
hsa-miR-570-4395458
hsa-miR-137-4373301
hsa-miR-486-3p-4395204
hsa-miR-518e-4395506
hsa-miR-92a-4395169
hsa-miR-383-4373018
hsa-miR-410-4378093
hsa-miR-204-4373094
hsa-miR-342-3p-4395371
hsa-miR-532-3p-4395466
hsa-miR-886-3p-4395305
hsa-miR-135a-4373140
hsa-miR-299-5p-4373188
hsa-miR-148a-4373130
hsa-miR-337-5p-4395267
hsa-miR-579-4395509
hsa-miR-491-5p-4381053
hsa-miR-423-5p-4395451
hsa-miR-19b-4373098
hsa-miR-30c-4373060
hsa-miR-211-4373088
hsa-miR-197-4373102
hsa-miR-499-5p-4381047
hsa-miR-510-4395352
hsa-miR-9-4373285
hsa-miR-509-5p-4395346
hsa-miR-519e-4395481
hsa-miR-302c-4378072
hsa-miR-489-4395469
hsa-miR-192-4373108
hsa-miR-215-4373084
hsa-miR-671-3p-4395433
hsa-miR-519d-4395514
hsa-miR-148b-4373129
hsa-miR-636-4395199
hsa-miR-217-4395448
hsa-miR-513-5p-4395201
hsa-miR-873-4395467
has-miR-155-4395459
hsa-miR-885-3p-4395483
hsa-miR-146a-4373132
hsa-miR-342-5p-4395258
hsa-miR-518d-3p-4373248
hsa-miR-373-4378073
hsa-miR-196b-4395326
hsa-miR-616-4395525
hsa-miR-208b-4395401
hsa-miR-33b-4395196
hsa-miR-876-5p-4395316
hsa-miR-889-4395313
hsa-miR-890-4395320
hsa-miR-891a-4395302
hsa-miR-871-4395465
hsa-miR-872-4395375
hsa-miR-875-3p-4395315
hsa-miR-876-3p-4395336
hsa-miR-654-3p-4395350
hsa-miR-654-5p-4381014
hsa-miR-672-4395438
hsa-miR-674-4395193
hsa-miR-624-4395541
hsa-miR-629-4395547
hsa-miR-651-4381007
hsa-miR-653-4395403
hsa-miR-582-5p-4395175
hsa-miR-589-4395520
hsa-miR-615-3p-4386777
hsa-miR-615-5p-4395464
hsa-miR-556-5p-4395455
hsa-miR-561-4380938
hsa-miR-576-3p-4395462
hsa-miR-576-5p-4395461
hsa-miR-548c-3p-4380993
hsa-miR-548d-3p-4381008
hsa-miR-551b-4380945
hsa-miR-556-3p-4395456
hsa-miR-544-4395376
hsa-miR-548a-3p-4380948
hsa-miR-548a-5p-4395523
hsa-miR-548b-3p-4380951
hsa-miR-525-3p-4395496
hsa-miR-525-5p-4378088
hsa-miR-526b-4395493
hsa-miR-541-4395312
hsa-miR-520d-5p-4395504
hsa-miR-520f-4373256
hsa-miR-520g-4373257
hsa-miR-524-5p-4395174
hsa-miR-518c-4395512
hsa-miR-518d-5p-4395500
hsa-miR-519c-3p-4373251
hsa-miR-520a-5p-4378085
hsa-miR-515-5p-4373242

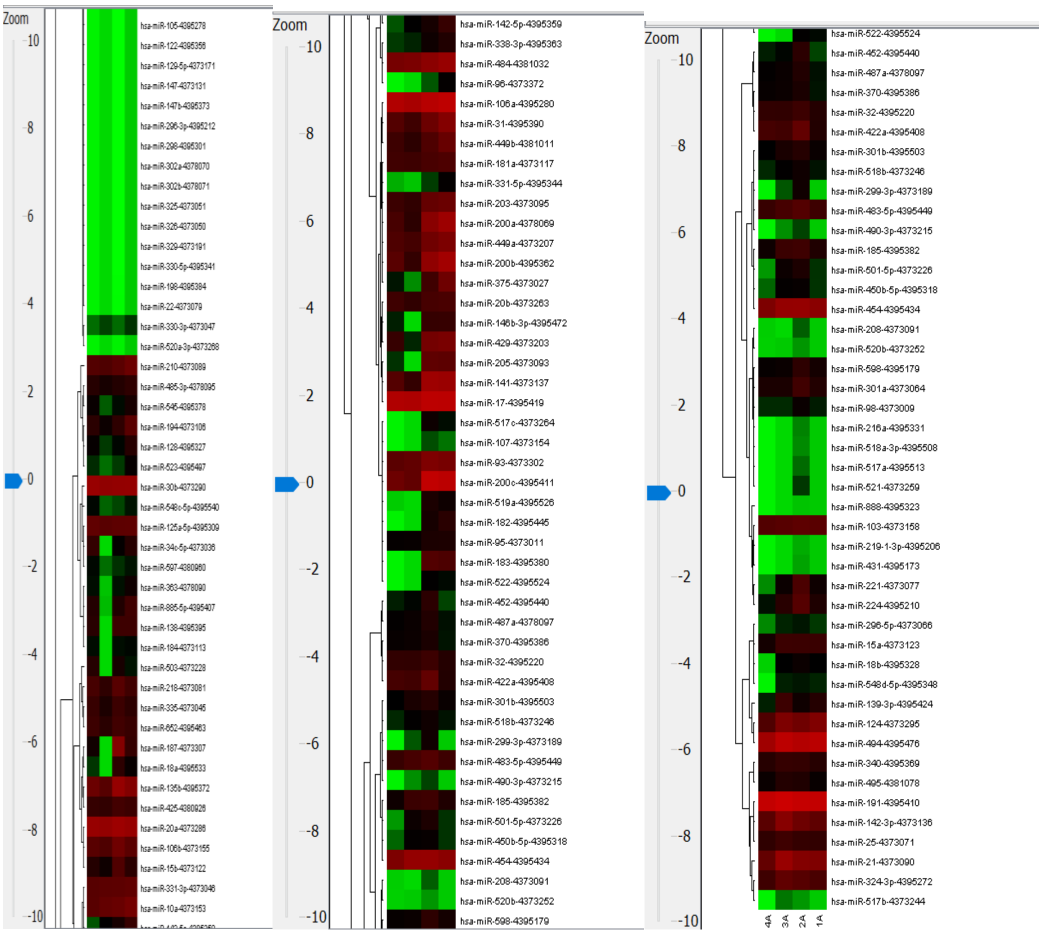

Figure S1a. Heat map of part 1 of the study – four cohorts (n = 20 samples in each cohort): OE, CG, and high-grade ovarian cancer and endometrioid ovarian cancer. Red indicates a decrease in the ΔC_T value (increased expression) and green indicates an increase in the ΔC_T value (decreased expression).

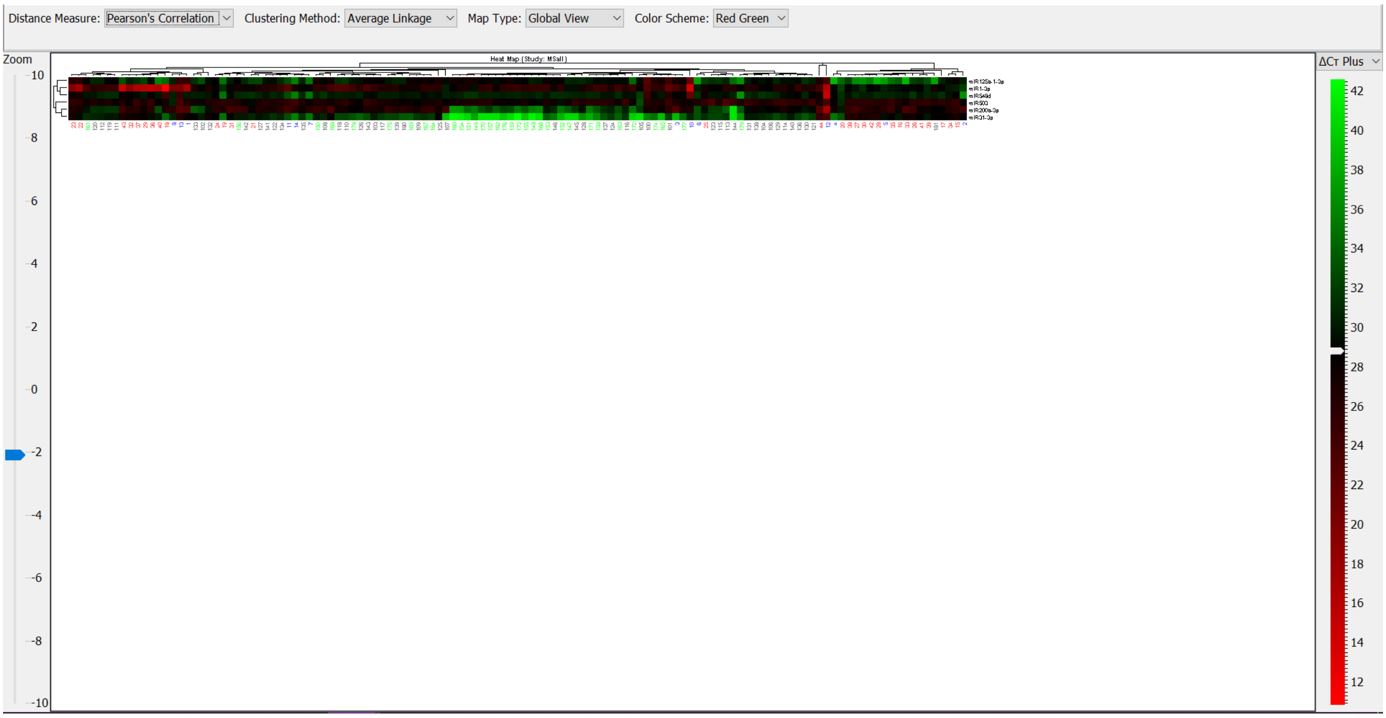

Figure S1b. Heat map of the 6 miRNAs with 2-fold differences in relative expression compared to the controls.
