# Supplementary material for: miR 31-3p Has the Highest Expression in Cesarean Scar Endometriosis
Source: Int J Mol Sci. 2022 Apr 22;23(9):4660. doi: 10.3390/ijms23094660 (PMC9105608; doi:10.3390/ijms23094660)
Supplement: Supplementary file 1 [file ijms-23-04660-s001.zip › ijms-1593377-supplementary/Supplementary File S3 - Correlations_revised.pdf]

Figure S2. Correlations.

In DIE miR-1-3p correlated positively with miR-125b-1-3p (R=0.738; p=0.037; Spearman correlation). In SE miR-125b-1-3p correlated positively with miR-548d (R=0.484; p=0.02) and miR-31-3p (R=0.417; p=0.043) and miR-200b-3p correlated positively with miR-31-3p (R=0.38; P=0.01). In OE miR-502 correlated positively with miR-503 (R=0.402, p=0.005) and miR-548d (R=0.527 , p=0.001).

DIE:

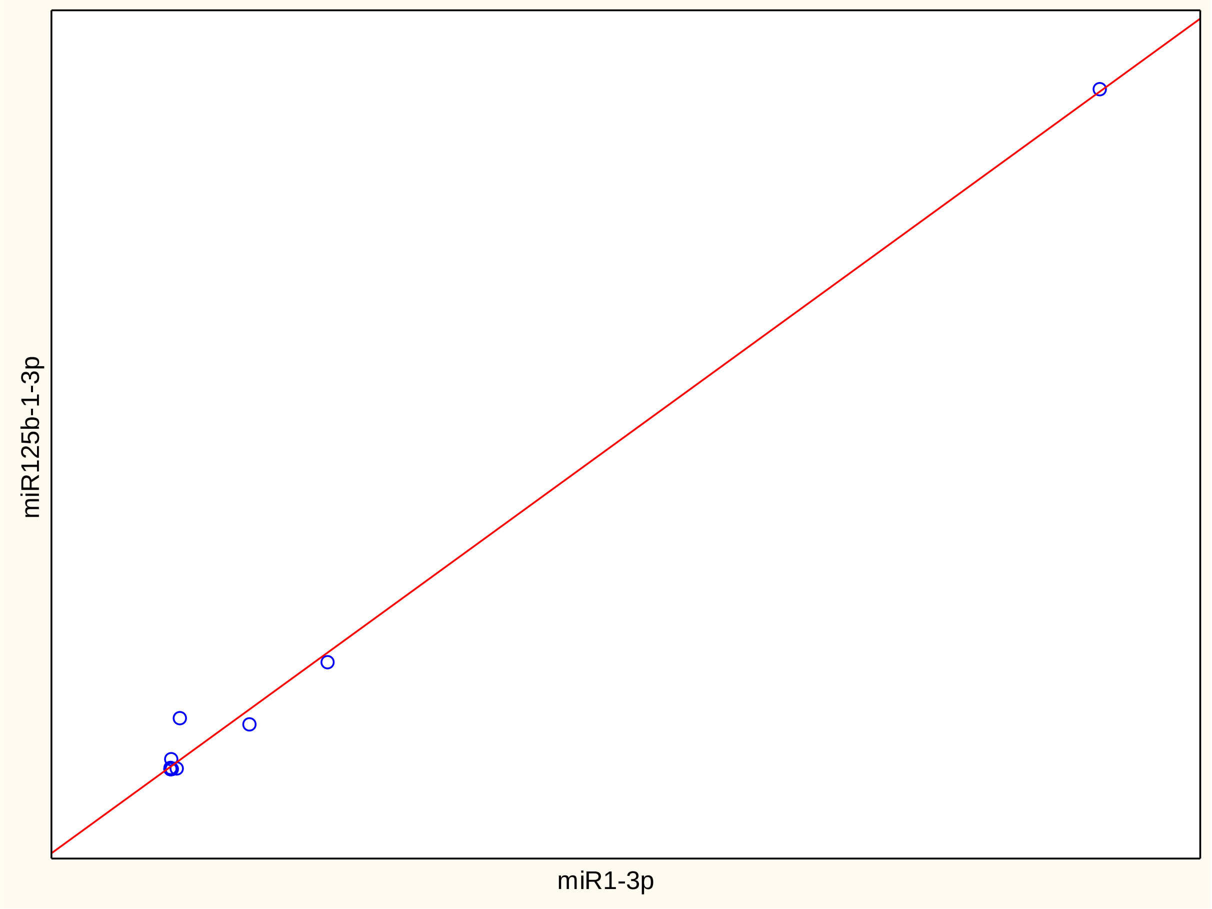

SE:

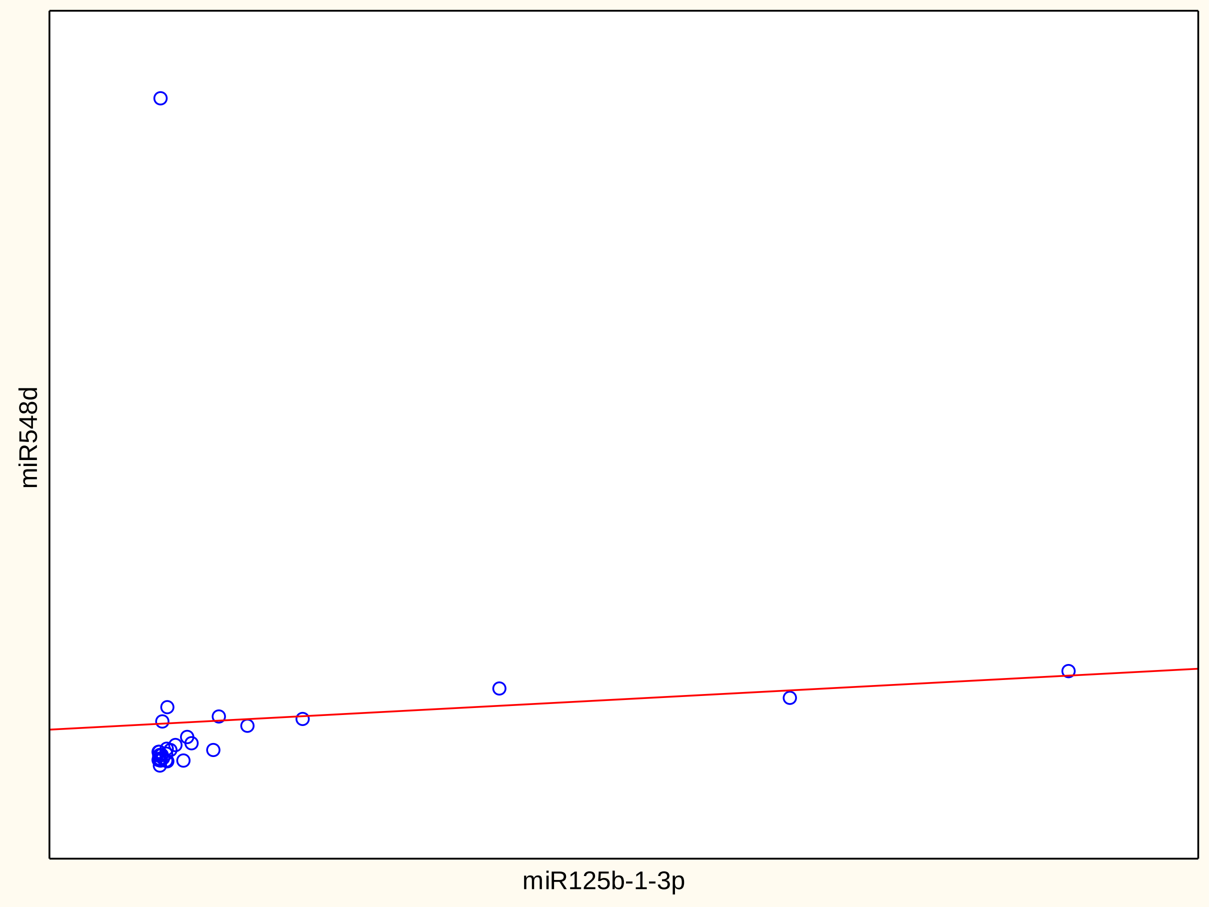

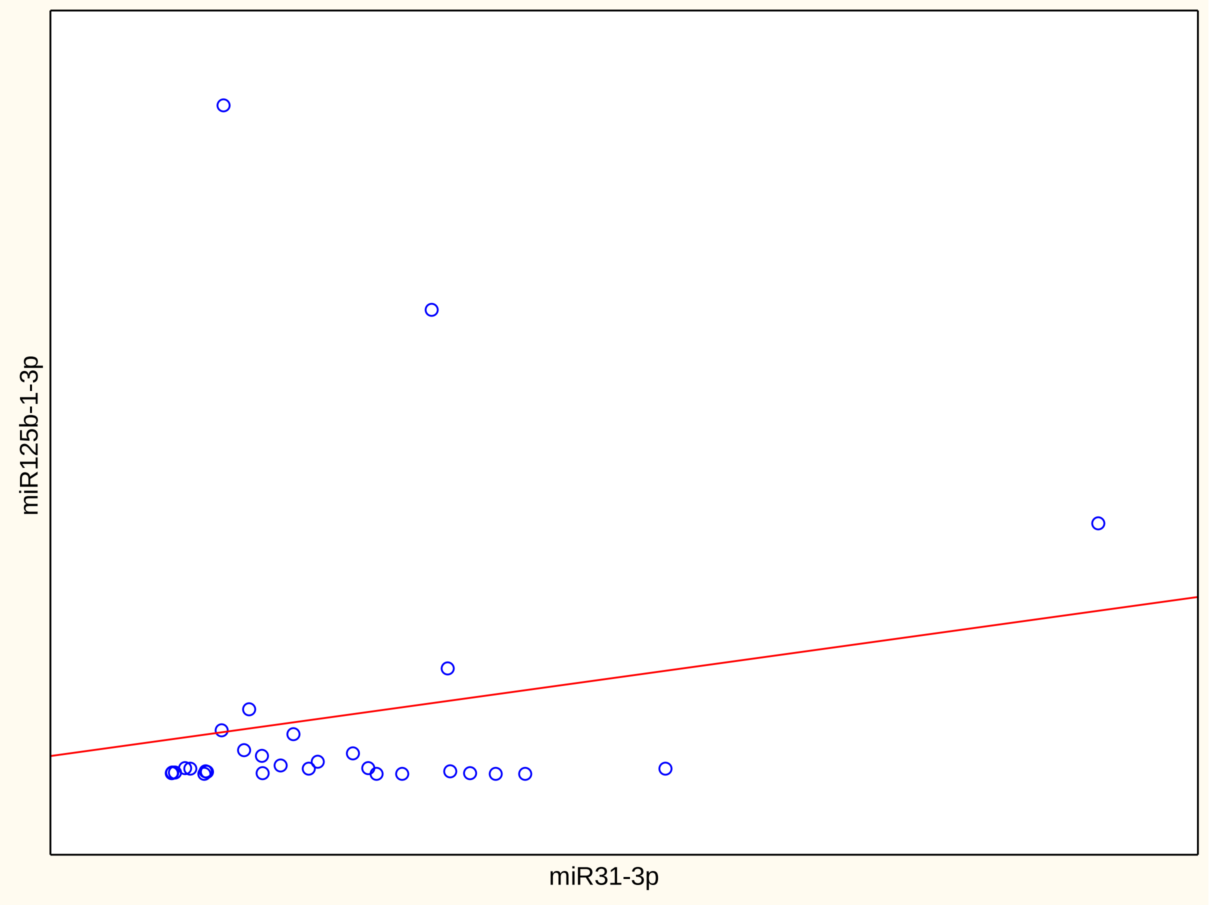

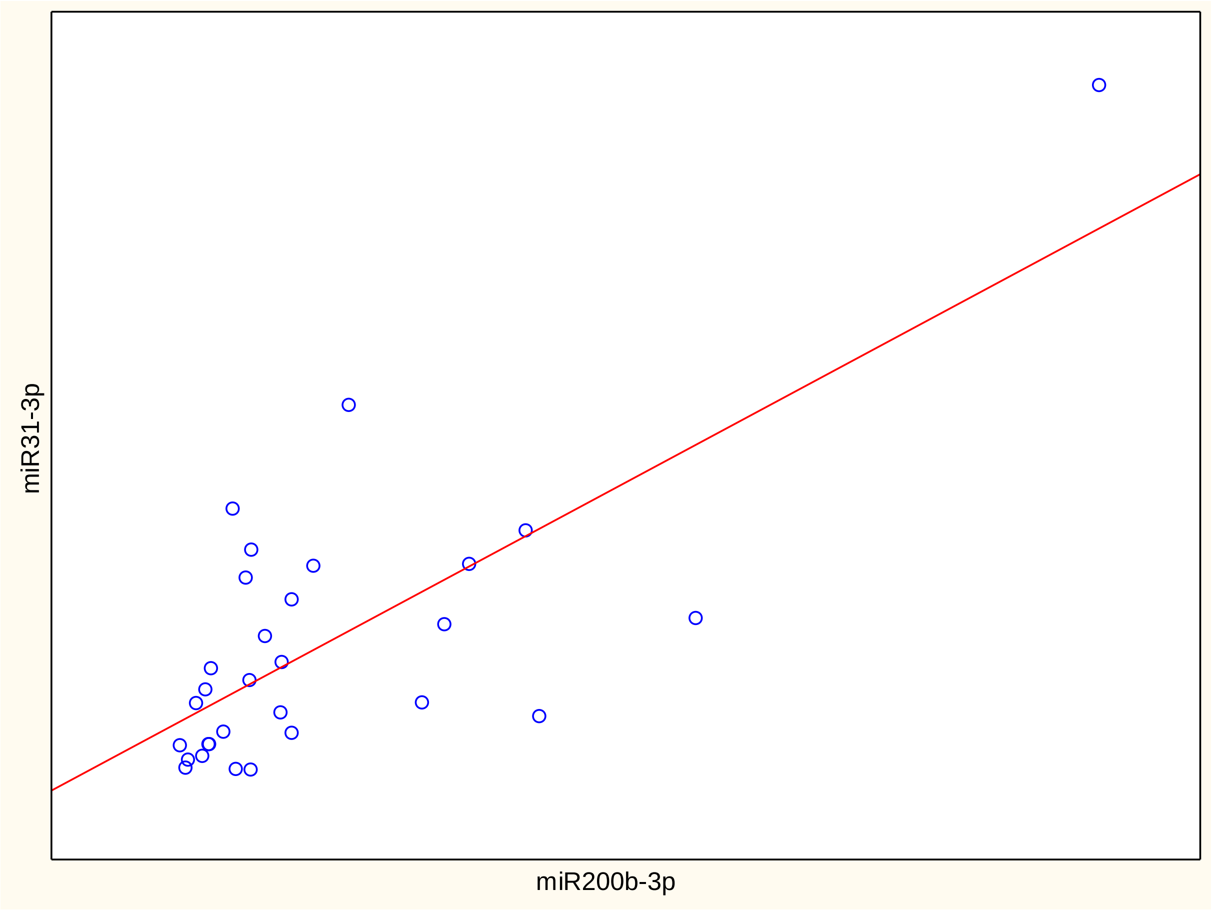
miR31-3p
miR200b-3p

OE:

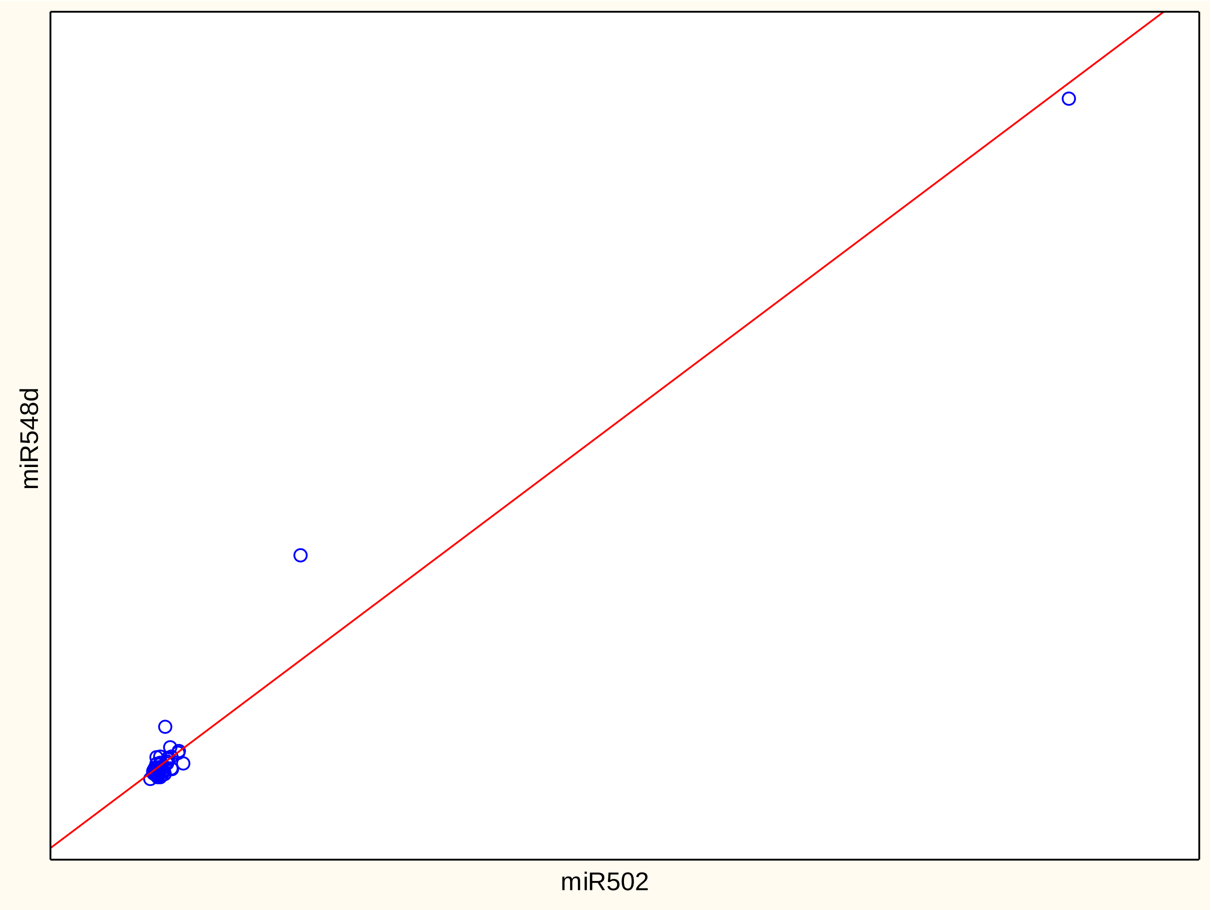

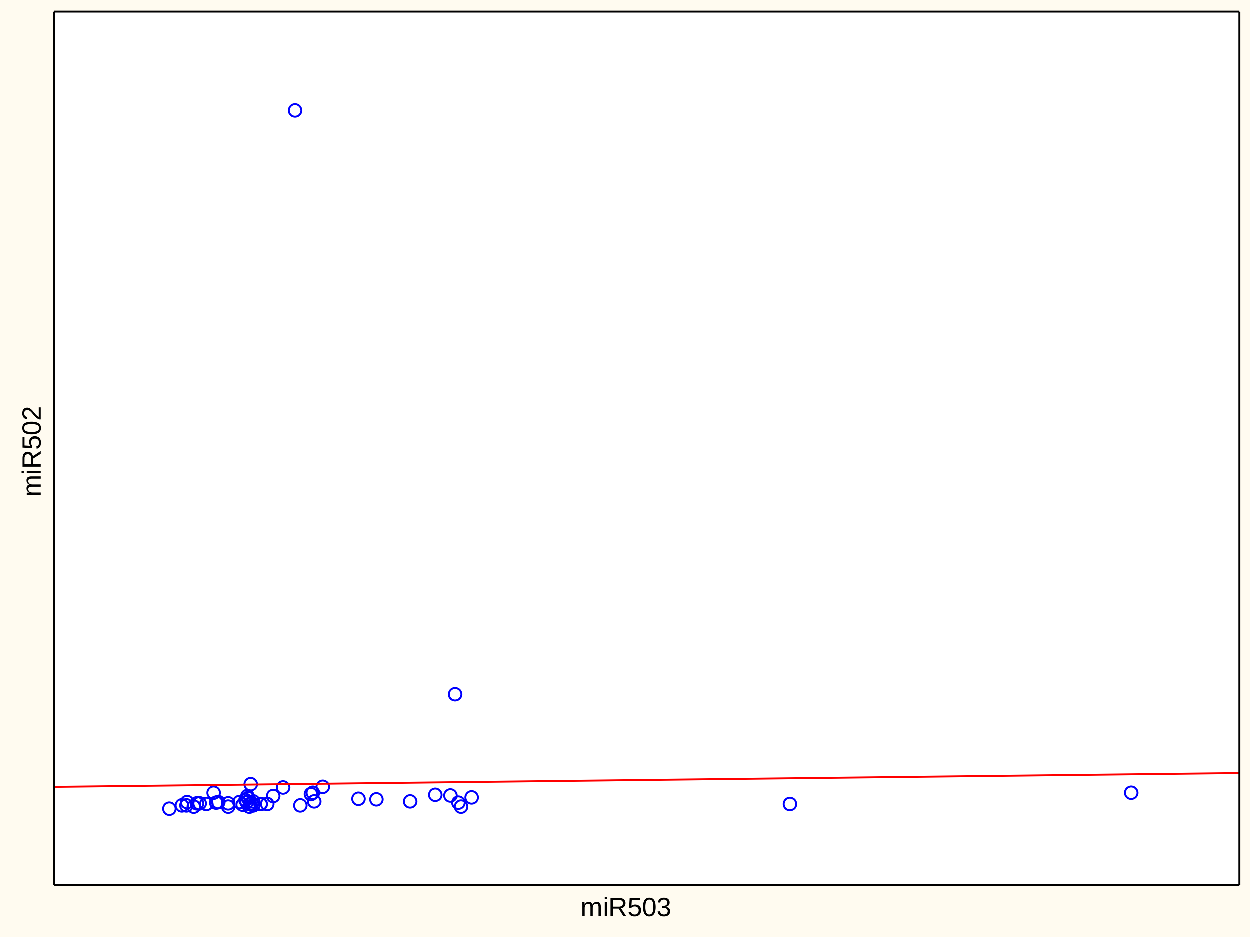

miR502
miR503
