# Supplementary material for: miR 31-3p Has the Highest Expression in Cesarean Scar Endometriosis
Source: Int J Mol Sci. 2022 Apr 22;23(9):4660. doi: 10.3390/ijms23094660 (PMC9105608; doi:10.3390/ijms23094660)
Supplement: Supplementary file 1 [file ijms-23-04660-s001.zip › ijms-1593377-supplementary/Supplementary File S4 - Population charateristics_revised.pdf]

	mean	median	min	max	SD
**Age [years]**					
DIE	36.28	33.5	25.0	57.0	9.425
OE	38.91	35.0	19.0	84.0	13.51
SE	36.367	36.0	26.0	48.0	5.61
CG	58.091	56.0	31.0	88.0	12.523
**BMI [kg/m^2]**					
DIE	23.321	23.6	18.7	32.5	3.446
OE	25.209	24.3	19.5	37.3	3.914
SE	27.743	24.95	18.4	38.5	14.342
CG	27.545	26.3	23.0	39.2	4.047
**Vaginal deliveries**					
DIE					
OE	0.714	0.5	0.0	3.0	0.914
SE	0.681	0.0	0.0	4.0	1.002
CG	0.133	0.0	0.0	1.0	0.346
	1.515	2.0	0.0	4.0	1.121
**Cesarean sections**					
DIE					
OE	0.357	0.0	0.0	2.0	0.663
SE	0.255	0.0	0.0	2.0	0.570
CG	1.43	1.0	0.0	3.0	0.679
	0.333	0.0	0.0	3.0	0.692
**Misscariages**					
DIE	0.357	0.0	0.0	3.0	0.842
OE	0.404	0.0	0.0	3.0	0.681
SE	0.4	0.0	0.0	2.0	0.675
CG	0.606	0.0	0.0	4.0	0.933

Characteristics of the studied population ($p<0.05$ for age between the control group (CG) and the endometriosis group (OE) – these difference could not be avoided due to ethical reasons.
